# Childhood trauma and the enduring consequences of forcibly separating children from parents at the United States border

**DOI:** 10.1186/s12916-018-1147-y

**Published:** 2018-08-22

**Authors:** Martin H. Teicher

**Affiliations:** 1 Department of Psychiatry, Harvard Medical School, Boston, USA; 2 Developmental Biopsychiatry Research Program, McLean Hospital, 115 Mill Street, Belmont, MA 02478 USA

**Keywords:** Childhood trauma, Early life stress, Refugees, Migrants, Family separation, Psychopathology, Sensitive periods, Brain development, Adverse childhood experiences

## Abstract

Forcible separation and detention of children from parents seeking asylum in the United States has been decried as immoral and halted by court order. Babies and children have been separated and transported to facilities sometimes many miles away. Limited data on forced detention of unaccompanied minors reveal high incidence of posttraumatic stress, anxiety disorders, depression, aggression, and suicidal ideation. These consequences will be magnified in youths forcibly separated from their parents, particularly younger children who depend on attachment bonds for self-regulation and resilience. Studies exploring the neuropsychiatric consequences of traumatic stress have revealed consistent effects of early life stress on brain structure, function and connectivity, and the identification of sensitive periods, which occur throughout childhood when specific regions and pathways are strongly influenced by adversity. Studies of epigenetics, inflammation and allostatic load are similarly enhancing our awareness of the molecular mechanisms underpinning the long-term consequences of traumatic stress. We must consider effects on the developing brain, mind and body to appreciate the long-term consequences of policies that force separation and detention of children.

## Background

The ‘zero-tolerance’ immigration policy initiated by the Trump administration led, within a matter of months, to the forced removal of thousands of children from their parents who were incarcerated for crossing the border in an effort to legally seek asylum. Politicians on both sides of the political spectrum, as well as the Pope, the Office of the United Nations High Commissioner for Human Rights, American Medical Association, American Academy of Pediatrics, and American Psychiatric Association decried this action. Bowing to intense pressure, President Trump amended this policy by executive order, hoping that law would be modified to enable children to be detained indefinitely with their families. Instead, on June 27, 2018, a federal judge denied this request and ordered US immigration agents to cease separating parents and children, and to reunite families split up within 30 days, and children younger than 5 within 14 days.

As health professionals, we need to understand the ramifications of separating children from their parents, and speak out against policies that so endanger the mental and physical wellbeing of children and families.

## Impact of forcible separation and traumatic stress

Some argue that actions taken by the US Government were relatively inconsequential to detained children because, as asylum-seeking immigrants, these children and their families were fleeing their home countries in a desperate effort to avoid more dire consequences such as violence, imminent threats of death or trafficking. However, this ignores the enormous protective power of families.

Children are dependent on the adults around them for their survival, and they can endure great hardship in the presence of parents with whom they feel protected and cared for. Forcibly removing a child from their parents is one of the most profound traumas a child can experience, since it undermines a pivotal foundation they require for self-regulation and resilience [1]. Similarly, having your children forcibly taken, not knowing where they are, and not being allowed to contact them, is many parents’ worst nightmare. Indeed, this is why members of the Trump administration advocated its use as a deterrent to immigration.

Some also question whether a few weeks or months of forced separation can have enduring effects. However, we know that brief traumatic events, such as being raped or witnessing violence to a loved one, can have life-long consequences [2]. Traumatic events are often betrayals of trust, or shatter our notions of safety and security. The impact of forced separation by the Trump administration will not end when children and parents are reunited. Many will live in fear that this will happen again, and this can have enduring epigenetic effects on the stress response system and attendant allostatic load – in turn increasing long-term risk for obesity, type 2 diabetes, chronic inflammation, and cardiovascular disease [3].

Clinically, evidence from studies of unaccompanied minors seeking asylum reveals that forced detention is associated with a high risk of posttraumatic stress disorder, anxiety disorder, depression, aggression, psychosomatic complaints, and suicidal ideation [4]. This is not surprising since adverse childhood experiences account for about 45% of the population-attributable risk for childhood onset psychiatric disorders [5]. One of the more disturbing features reported about the detention centers was their rule forbidding children from touching or hugging each other, including siblings.

## Sensitive exposure periods and brain development

Nursing infants, toddlers, youths and teens have all been removed from their parents, and many will suffer a variety of age-specific psychiatric and neurobiological effects as a result. The central nervous system undergoes profound maturational changes during all stages of childhood, and various brain regions and pathways have their own unique sensitive periods during which experience can most dramatically shape and fine-tune their synaptic structure and interconnections. Studies in my laboratory and other centers have begun to identify the developmental stages at which specific structures are most vulnerable.

Regions especially susceptible to stress during the first seven postnatal years [6–10] are involved in detecting and responding to threats, and in the regulation of stress response. Modifying this system is one of the primary ways our brains are shaped by early adversity [11]. These regions are also involved in aspects of attention and memory, and these processes appear to be particularly vulnerable to adversity during early childhood [12].

Myelinating fiber pathways [13–15] and corticolimbic structures [16, 17] appear to be especially vulnerable during middle childhood. These pathways are critical for left–right hemispheric integration and sensory processing [11]. Which sensory systems, if any, may be affected by forced separation and detention, are not known but would likely depend on aspects of detention that children found most aversive. For example, in previous research by our laboratory, physical and emotional neglect at 11 years of age emerged as a key determinant of social cognition [12].

Brain regions affected by adversity during the peripubertal and teenage years are involved in emotional regulation, impulse control, and other executive functions [7, 13, 18]. Adversity is also associated with significant alterations in brain network organization, primarily through effects on late-maturing association pathways [19]. Emotional abuse around the age of 15 emerged in another of our studies as the most important predictor of risk for major depression [20].

## Psychopathology with and without early life stress

Psychiatric disorders in individuals exposed to severe childhood stress have an earlier age of onset, more comorbidities, and a more severe course and poorer response to first line treatments than in unexposed individuals with the same primary psychiatric diagnosis [21]. They also have an array of neurobiological alterations [11] and signs of chronic inflammation [22] not found in their unexposed counterparts, which has led us to propose that psychiatric disorders presenting in individuals with early life stress represent a unique and clinically challenging ecophenotype (i.e., a modified phenotype resulting from environmental influences) [21]. Hence, children separated from families seeking asylum may be further burdened with difficult-to-treat disorders that emerge years later, particularly as they pass through puberty [11].

## Conclusions

Safe, supportive and nurturing relationships with primary caregivers are critical for the healthy physical and emotional development of children. Parents play an essential role in enabling children already exposed to serious adversity to cope and effectively recover by buffering their stress response, facilitating their ability to self-regulate, and helping them to rebuild a sense of security [1]. Removing children from their parents is a governmental prerogative that should only be used after judicial review, and when necessary to protect children from harm from abusive or neglectful parents.

It is sad to note that the United States implemented this inhumane policy, and is the only member country of the UN that has not ratified the United Nations Convention on the Rights of the Child. Leaders who advocated and advanced a policy tantamount to state sponsored child abuse should be held accountable. Anyone callous enough to treat children of refugees in this way can hardly be trusted to treat other people’s children, even those of American citizens, in a wise and caring manner. Fortunately, recordings of inconsolable children crying released in the media touched enough hearts to show that there is a majority of Americans who care about children. Societies reap what they sow in terms of the way they treat their children – as well as the children of brave and resourceful asylum seekers eager to become part of the American experience. A nation striving for greatness would do well to keep this in mind.
